# Metabolic Dysfunction‐Associated Steatotic Liver Disease and Obesity: Pathogenesis, Diagnostics, Risk Stratification, and Therapeutic Approach

**DOI:** 10.1002/kjm2.70227

**Published:** 2026-04-24

**Authors:** Beom Kyung Kim

**Affiliations:** ^1^ Department of Internal Medicine Yonsei University College of Medicine Seoul Republic of Korea; ^2^ Institute of Gastroenterology Yonsei University College of Medicine Seoul Republic of Korea; ^3^ Yonsei Liver Center, Severance Hospital Yonsei University Health System Seoul Republic of Korea

**Keywords:** metabolic dysfunction‐associated steatotic liver disease, obesity, pathogenesis, therapy

## Abstract

Metabolic dysfunction‐associated steatotic liver disease (MASLD) has emerged as the most prevalent chronic liver disease worldwide, closely linked to the global rising incidence of obesity and metabolic syndrome. This review synthesizes current evidence on the pathogenesis, gut–liver axis, and multidisciplinary management of MASLD within the context of obesity. The pathophysiology of MASLD is multifactorial and involves insulin resistance, adipose tissue dysfunction, chronic low‐grade inflammation, and alterations in lipid metabolism, all of which contribute to hepatic steatosis and disease progression. Recent research has increasingly focused on the gut–liver axis, where dysbiosis, increased intestinal permeability, endotoxemia, and microbial metabolites significantly influence hepatic inflammation and fibrogenesis. From a therapeutic perspective, lifestyle modifications remain foundational to management; however, their long‐term sustainability is often limited. Pharmacologic interventions targeting metabolic pathways, such as incretin‐based therapies, demonstrate promising efficacy in enhancing both hepatic and systemic outcomes. Given the substantial clinical and socioeconomic burden posed by MASLD, a multidisciplinary approach that integrates perspectives from hepatology, endocrinology, nutrition, and public health is essential. Future research should prioritize personalized risk assessment, early intervention in high‐risk populations, and policy‐level strategies to mitigate the growing impact of metabolic liver disease.

## 1 Introduction, Global Epidemiology, and Nomenclature Evolution

Metabolic dysfunction–associated steatotic liver disease (MASLD) has solidified its status as the most prevalent chronic liver disease worldwide, affecting an estimated 25%–38% of the global adult population [1, 2, 3]. Given the disease's significant association with systemic metabolic syndrome [4], a multisociety Delphi consensus involving experts from hepatology, endocrinology, and obesity has recently led to a substantial paradigm shift in disease nomenclature [5]. The previously used terms “nonalcoholic fatty liver disease” (NAFLD) and “non‐alcoholic steatohepatitis” (NASH) have been retired due to their imprecision, reliance on exclusionary diagnostics, and the stigma associated with the terms “non‐alcoholic” and “fatty” [5].

Under the newly established framework, steatotic liver disease serves as the overarching umbrella term [5]. MASLD is definitively diagnosed by the presence of hepatic steatosis (quantified via imaging or biopsy) alongside at least one of five distinct cardiometabolic risk factors: (1) elevated body mass index (BMI ≥ 25 kg/m^2^, or ≥ 23 kg/m^2^ in Asian populations) or increased waist circumference (> 94 cm in men, > 80 cm in women); (2) impaired fasting glucose (≥ 5.6 mmol/L), elevated HbA1c (≥ 5.7%), or overt Type 2 diabetes mellitus (T2DM); (3) hypertension (blood pressure ≥ 130/85 mmHg or specific antihypertensive pharmacological treatment); (4) hypertriglyceridemia (≥ 1.70 mmol/L); and (5) low high‐density lipoprotein cholesterol (≤ 1.0 mmol/L in males, ≤ 1.3 mmol/L in females).

Importantly, the updated consensus has also introduced the subclassification “MetALD” to accurately characterize individuals who meet the diagnostic criteria for MASLD while simultaneously consuming moderate to high amounts of alcohol (defined as 140–350 g/week for females and 210–420 g/week for males) [5]. This classification addresses a critical historical oversight, as patients with both metabolic risk factors and moderate alcohol intake were previously excluded from clinical trials despite facing a markedly higher risk of adverse liver‐related and overall mortality. The progressive, necroinflammatory stage of the disease, formerly known as NASH, has been aptly renamed metabolic dysfunction‐associated steatohepatitis (MASH). As MASLD coexists in over 70% of obese adults with T2DM, addressing excess adiposity and the resultant insulin resistance remains the cornerstone of clinical management [6, 7, 8, 9, 10].

## 2 Molecular Pathogenesis: The Multi‐Hit Hypothesis and Lipotoxicity

The pathophysiological transition from isolated, benign hepatic steatosis to necroinflammatory MASH and advanced fibrosis is best explained by the “multiple hit” hypothesis [2, 11]. This model suggests a synergistic and catastrophic convergence of systemic insulin resistance, profound lipotoxicity, endoplasmic reticulum (ER) stress, oxidative damage, and severe gut dysbiosis [12, 13, 14].

### 2.1 Systemic Insulin Resistance and Adipose Tissue Dysfunction

Systemic insulin resistance (IR) serves as the primary pathophysiological driver initiating the MASLD cascade [15]. In a healthy physiologic state, insulin binds to the alpha and beta subunits of the insulin receptor, which recruits substrates like insulin receptor substrates (IRS‐1 and IRS‐2) to suppress lipolysis in white adipose tissue and reduce hepatic gluconeogenesis while promoting glucose uptake in skeletal muscle via GLUT4 transporters [16]. However, in the context of chronic overnutrition and significant obesity, adipocytes undergo severe hypertrophy, leading to localized cellular hypoxia and the recruitment of pro‐inflammatory M1‐polarized macrophages into the adipose tissue. These activated M1 macrophages secrete large quantities of pro‐inflammatory cytokines, including interleukin‐6 and tumor necrosis factor‐alpha, which directly antagonize IRS signaling. This antagonism precipitates unregulated and catastrophic lipolysis, resulting in a substantial efflux of free fatty acids that are directed via the portal circulation into the liver [17]. Moreover, hypertrophic adipocyte dysfunction alters the expression of key adipokines, significantly increasing leptin levels (leading to leptin resistance) while critically decreasing adiponectin, a hormone that typically promotes fatty acid β‐oxidation and suppresses hepatic lipid synthesis [18].

### 2.2 Hepatic Lipotoxicity, Organelle Stress, and Fibrogenesis

When hepatic lipid uptake and carbohydrate‐driven de novo lipogenesis (DNL)—heavily fueled by fructose consumption—exceed the liver's maximum capacity for mitochondrial beta‐oxidation and very‐low‐density lipoprotein (VLDL) exportation, highly lipotoxic intermediates accumulate [19]. Specifically, diacylglycerols (DAGs) and ceramides accumulate within the hepatocyte cytoplasm. DAGs activate protein kinase C epsilon, which directly phosphorylates and critically impairs IRSs (particularly at the Thr1160 residue of the insulin receptor kinase), exacerbating hepatic IR in a vicious, self‐amplifying cycle [20]. Paradoxically, this selective hepatic IR diminishes insulin's capability to suppress gluconeogenesis, while the DNL pathway remains uninhibited and hyperactive due to the activation of sterol regulatory element‐binding protein 1c (SREBP‐1c) through the mTOR pathways [21].

This profound lipotoxic burden induces severe ER stress, triggering the unfolded protein response mediated by transmembrane sensors such as IRE1, PERK, and ATF6. Simultaneously, mitochondrial dysfunction leads to the excessive production of reactive oxygen species (ROS), resulting in hepatocyte ballooning, apoptosis, and inflammasome activation [22]. Hepatocellular death subsequently recruits inflammatory cells and activates quiescent hepatic stellate cells (HSCs). HSCs undergo transdifferentiation into highly proliferative, contractile myofibroblasts, shifting from the production of typical Type IV collagen to the deposition of rigid Type I and III collagens, thereby cementing the progression of hepatic fibrosis [23].

### 2.3 Sarcopenic Obesity and Muscular Crosstalk

Skeletal muscle plays a crucial role in the disposal of approximately 80% of postprandial glucose through insulin‐mediated mechanisms [24]. In obese individuals, the accumulation of ectopic intramuscular lipids, specifically lipotoxic DAGs and ceramides, severely disrupts insulin signaling by downregulating GLUT4 transporters, which drastically reduces glucose uptake and diminishes glycogen synthesis [25]. This diminished metabolic capacity compels additional unoxidized glucose and lipids to be directed toward the liver. Increased fatty acid beta‐oxidation in muscle tissues elevates ROS production, leading to myocyte toxicity and accelerating muscle atrophy (i.e., sarcopenia) [26]. This bidirectional failure significantly exacerbates sarcopenic obesity, a condition that poses a markedly heightened risk for the progression to severe hepatic fibrosis and increased overall mortality in cohorts with MASLD.

### 2.4 Genetic, Epigenetic, and Environmental Determinants

The genetic landscape plays a significant role in modulating individual susceptibility to MASLD and influences the rate of fibrotic progression [27, 28]. Polymorphisms in the *PNPLA3* gene (particularly the I148M variant, which is strongly enriched in Hispanic populations) result in a protein that is resistant to ubiquitylation and proteasomal degradation [29]. This resistance leads to marked hepatic triglyceride accumulation and directly promotes fibrogenesis through enhanced HSC activation. Similarly, the *TM6SF2* variant (E167K) critically impairs the export of hepatic VLDL, which physically traps lipids within the hepatic parenchyma while paradoxically conferring a protective cardiovascular lipid profile [30]. Additionally, single‐nucleotide polymorphisms in the *MBOAT7* and *GCKR* genes further increase the risk of steatosis [31]. Conversely, loss‐of‐function variants in *HSD17B13* have been shown to provide robust protection against chronic liver injury and advanced MASH independent of systemic IR [32]. Beyond genetic factors, environmental exposures to endocrine‐disrupting chemicals, such as per‐ and polyfluoroalkyl substances, have been proven to profoundly disrupt hepatic lipid metabolism and exacerbate the severity of MASLD independently of caloric intake [33].

## 3 The Gut–Liver Axis, Microbiome Dysbiosis, and Immune Crosstalk

The gastrointestinal tract and liver communicate extensively through the biliary tract and portal circulation, establishing a highly complex and bidirectional gut–liver axis [12]. Dysbiosis, often characterized by an altered *Firmicutes*/*Bacteroidetes* ratio and significantly reduced overall microbial diversity, is central to the pathogenesis of MASH [34].

### 3.1 Intestinal Permeability and Endotoxemia

Obesity and high‐fat Western diets significantly compromise the integrity of tight junctions in the intestinal epithelium and degrade the protective mucin layer, leading to increased paracellular permeability, commonly referred to as “leaky gut.” [35] This structural disruption allows pathogen‐associated molecular patterns, particularly lipopolysaccharides (LPS) derived from the cell walls of gram‐negative bacteria, to translocate directly into the portal vein. Once in the liver, LPS binds robustly to Toll‐like receptor 4 on resident Kupffer cells, initiating a substantial pro‐inflammatory cascade mediated by NF‐κB and the NLRP3 inflammasome [36]. This immune activation heavily promotes the critical transition from simple steatosis to necroinflammatory MASH. Furthermore, specific gut microbes, such as *Escherichia* and *Klebsiella pneumoniae* , can metabolize dietary carbohydrates to produce endogenous ethanol, subjecting the liver to significant oxidative stress akin to that observed in alcoholic liver injury, even in patients who abstain from alcohol [37, 38].

### 3.2 Bile Acid Metabolism and Nuclear Receptor Signaling

Bile acids (BAs) serve as essential signaling molecules that regulate lipid and glucose homeostasis through the activation of the Farnesoid X Receptor (FXR) and the Takeda G‐protein‐coupled receptor 5 (TGR5) [39, 40]. Primary BAs (e.g., cholic acid, chenodeoxycholic acid) are synthesized de novo in the liver from cholesterol and undergo extensive microbial modification (deconjugation and dehydroxylation) via bile salt hydrolases to form secondary BAs (e.g., deoxycholic acid and lithocholic acid) within the intestine. Dysbiosis significantly alters this BA pool, disrupting the FXR and TGR5 signaling. Impaired activation of FXR fails to suppress hepatic CYP7A1, leading to unregulated BA synthesis while also failing to inhibit hepatic de novo lipogenesis mediated by SREBP‐1c, thereby exacerbating lipotoxicity and systemic inflammation [41]. Conversely, robust activation of TGR5 in intestinal L‐cells stimulates the secretion of glucagon‐like peptide‐1 (GLP‐1), improving insulin sensitivity [42].

### 3.3 Microbial Metabolites: Short‐Chain Fatty Acids (SCFAs) and Trimethylamine *N*‐Oxide (TMAO)

SCFAs, such as butyrate, acetate, and propionate, produced through the microbial fermentation of indigestible dietary fiber, exert significant anti‐inflammatory effects via G‐protein‐coupled receptors like GPR43 and GPR41 [43]. However, in MASLD, dysbiosis also facilitates the detrimental conversion of dietary choline into trimethylamine (TMA) by gut bacteria [44]. The liver subsequently oxidizes TMA through the enzyme FMO3 to form TMAO, a highly toxic metabolite closely linked to atherosclerosis, severe cardiovascular disease, and advanced MASH severity. Alterations in the microbiome consistently reveal an increased relative abundance of *Proteobacteria*, *Prevotella*, and *Streptococcus*, accompanied by critical depletions of beneficial SCFA‐producing genera, such as *Faecalibacterium*, *Coprococcus*, and *Ruminococcaceae* [45].

### 3.4 The Mycobiome and Virome

Emerging evidence underscores the significant roles of the gut mycobiome and virome in the pathogenesis of MASLD. An overrepresentation of opportunistic fungi such as *Candida albicans* and *Mucor* species has been documented in patients with MASH [46]. Fungal cell wall components, particularly β‐glucans, powerfully engage the C‐type lectin receptor Dectin‐1 on hepatic Kupffer cells, triggering intense NF‐κB signaling and NLRP3 inflammasome activation [47]. Additionally, advanced NAFLD is associated with a severe reduction in intestinal viral diversity, particularly a loss of gut bacteriophage richness, which typically regulates pathogenic gram‐negative bacterial populations.

## 4 Noninvasive Diagnostics and Risk Stratification

Early‐stage MASLD is predominantly asymptomatic; therefore, proactive clinical risk stratification is essential for identifying patients at high risk for advanced fibrosis (F3–F4). This risk remains the most critical predictor of liver‐related mortality and decompensation [48, 49, 50, 51].

### 4.1 Noninvasive Serum Tests (NITs)

The fibrosis‐4 (FIB‐4) index, which incorporates the patient's age, aspartate aminotransferase (AST), alanine aminotransferase (ALT), and platelet count, is universally recognized by hepatology guidelines as the primary first‐line screening tool. A FIB‐4 score < 1.3 robustly excludes advanced fibrosis, whereas a score ≥ 2.67 (or > 2.0 in adults > 65 years) necessitates immediate secondary noninvasive assessments or urgent referral to hepatology [52]. However, the FIB‐4 index has an indeterminate “gray zone” (1.3–2.67) and may produce high false‐negative rates in younger obese patients or those with severe T2DM [53, 54]. The NAFLD Fibrosis Score serves as an alternative serum‐based stratification tool that utilizes age, BMI, dysglycemia, AST/ALT ratio, platelets, and albumin [55].

### 4.2 Advanced Elastography

Vibration‐controlled transient elastography (commercially known as FibroScan) is the preferred second‐line imaging modality for measuring liver stiffness [48]. A liver stiffness measurement (LSM) < 8.0 kPa generally rules out advanced fibrotic disease, while an LSM ≥ 8.0 kPa indicates clinically significant fibrosis. LSM values > 15 kPa strongly suggest compensated advanced chronic liver disease, and scores > 25 kPa indicate a high risk for clinically significant portal hypertension requiring beta‐blocker (e.g., carvedilol) prophylaxis and hepatocellular carcinoma surveillance [56].

### 4.3 Magnetic Resonance Imaging‐Proton Density Fat Fraction (MRI–PDFF)

MRI–PDFF offers highly precise, noninvasive quantification of hepatic triglyceride content. It is particularly sensitive to dynamic changes in liver fat, making it the premier imaging biomarker for monitoring therapeutic efficacy in ongoing clinical trials [56].

### 4.4 Liver Biopsy

Despite liver biopsy being the gold standard for diagnosing steatohepatitis (assessing ballooning and lobular inflammation) and staging fibrosis from F0 to F4 [57], its inherent invasiveness, associated risk of bleeding complications, high sampling error rate, and considerable cost limit its routine application to complex clinical scenarios where NITs yield conflicting results or when coexisting etiologies are suspected.

## 5 Non‐Pharmacological Management: Dietary and Lifestyle Interventions

Intensive lifestyle modification remains the foundation of MASLD management, demonstrating a clear dose–response efficacy [58].

### 5.1 Caloric Deficit and Weight Loss Targets

To significantly reduce hepatic steatosis, a total body weight loss (TBWL) of ≥ 5% is essential, while a TBWL of ≥ 7%–10% is typically required to completely resolve necroinflammatory steatohepatitis and promote structural regression of fibrosis [1]. A recent study involving patients with T2DM–MASLD utilizing digital monitoring showed that a TBWL ≥ 5% resulted in a 2.38% absolute reduction in MRI–PDFF alongside a substantial 31.2% decrease in the Homeostatic Model Assessment of IR, underscoring the profound metabolic reset triggered by sustained weight loss [59].

### 5.2 Macronutrient and Micronutrient Modulation

The quality of the diet has a significant biological impact beyond mere caloric restriction.

- *Macronutrients:* An overabundance of saturated fatty acids, commonly found in processed meats and dairy products, leads to a pronounced increase in plasma ceramides, induces severe ER stress, and triggers lipotoxicity [60]. Conversely, monounsaturated fatty acids (MUFAs)—which are prevalent in olive oil and avocados—improve insulin sensitivity and mitigate lipotoxicity [61]. Omega‐3 polyunsaturated fatty acids (PUFAs) possess potent anti‐inflammatory and triglyceride‐lowering properties. Refined carbohydrates, particularly fructose (abundant in high‐fructose corn syrup), must be strictly avoided [62], as fructose is exclusively metabolized by the liver, resulting in excessive hepatic DNL, significant depletion of hepatic ATP, increased uric acid levels, and pronounced gut dysbiosis. High consumption of animal protein (e.g., red/processed meats) exacerbates IR, whereas plant‐based proteins, fish, and eggs provide essential choline, which is crucial for the exportation of VLDL [63].
- *Micronutrients and Bioactives:* Oxidative stress underscores the importance of antioxidant therapy. In the landmark PIVENS trial, vitamin E (800 IU/day) was shown to significantly improve steatosis and lobular inflammation in nondiabetic adults with biopsy‐confirmed MASH [64]. Deficiencies in vitamin D worsen IR via impaired binding of the vitamin D receptor on pancreatic beta cells, while deficiencies in vitamin B12 and folate (B9) are associated with increased severity of MASH [65]. Herbal supplements such as silymarin (milk thistle), curcumin (turmeric), and garlic extract (S‐allyl cysteine) modulate the NF‐κB pathways, leading to reduced inflammatory AST/ALT levels [66, 67, 68]. Moderate coffee consumption (2–3 cups/day) provides substantial hepatoprotection, demonstrating an inverse, dose‐dependent relationship with advanced fibrosis and hepatocellular carcinoma risk, attributed to its high polyphenol content [69].
- *Dietary Patterns:* The Mediterranean diet, which is rich in MUFAs, PUFAs, and prebiotic fiber, offers profound anti‐inflammatory benefits independent of substantial weight loss and is highly recommended as a dietary framework [70]. The DASH also contributes to a reduced metabolic risk [71]. Intermittent fasting promotes metabolic switching toward fatty acid oxidation and ketogenesis, yielding rapid improvements in hepatic steatosis when sustained over the long term [72].

### 5.3 Exercise Physiology and Circadian Rhythms

Both aerobic exercise and resistance training effectively reduce hepatic fat by enhancing mitochondrial oxidative capacity and markedly increasing GLUT‐4 expression in skeletal muscle, independent of substantial weight loss [73]. Clinical guidelines universally advocate for 150–300 min of moderate‐intensity or 75–150 min of vigorous‐intensity aerobic activity every week [74]. Resistance training is particularly crucial for combating sarcopenia, maintaining lean muscle mass, and preserving insulin‐mediated glucose metabolic flexibility in older or frail individuals. Exercise promotes the upregulation of PPAR‐γ and PGC1‐α, as well as the release of beneficial myokines such as irisin, which have direct anti‐steatogenic effects on the liver [75]. Furthermore, addressing disruptions in circadian rhythms—such as “social jetlag” and sleep fragmentation—is vital, as misaligned circadian cycles directly enhance the expression of lipogenic genes and worsen steatosis [76].

## 6 The Rapidly Evolving Landscape of Pharmacotherapy

For patients who are unable to achieve therapeutic targets through lifestyle interventions alone, or for those who present with clinically significant fibrosis (≥ F2), a rapidly expanding array of advanced pharmacotherapies has become available.

### 6.1 Incretin‐Based Therapies

Glucagon‐like peptide‐1 receptor agonists (GLP‐1 RAs), such as liraglutide and semaglutide, exert their effects centrally on the hypothalamus, inducing significant satiety and significantly delaying gastric emptying, thereby promoting a TBWL of 10%–15% [77]. The landmark LEAN trial demonstrated that liraglutide successfully achieves MASH resolution [78]. In extensive Phase 2 and 3 trials (e.g., the ESSENCE trial), semaglutide (at doses up to 2.4 mg weekly) significantly resolved steatohepatitis and rapidly decreased ALT and MRI–PDFF scores [79]. While the presence of direct GLP‐1 receptors on hepatocytes remains a topic of debate, the hepatic benefits are likely mediated indirectly through substantial systemic weight loss, greatly enhanced insulin sensitivity and paracrine signaling from local Kupffer and stellate cells [80]. Furthermore, oral GLP‐1 RAs, such as orforglipron and danuglipron, are currently demonstrating comparable efficacy in weight loss to their injectable counterparts, representing promising alternatives for the future [81].

### 6.2 Dual and Triple Incretin Agonists

Tirzepatide, a dual glucose‐dependent insulinotropic polypeptide (GIP)/GLP‐1 RA, has demonstrated overwhelming superiority over selective GLP‐1 RAs, achieving a TBWL > 15%–20% [82]. The addition of GIP agonism enhances nutrient partitioning in adipose tissue and effectively reduces visceral fat. The SURPASS‐3 MRI trial confirmed significant hepatic fat clearance alongside notable improvements in insulin sensitivity [83]. Retatrutide, an innovative investigational triple agonist targeting GLP‐1, GIP, and glucagon receptors, has demonstrated unprecedented weight loss (up to 24.2% in Phase 2 trials) and substantial reductions in liver fat [84]. The critical inclusion of glucagon receptor agonism specifically enhances hepatic beta‐oxidation and significantly increases resting energy expenditure, rendering it uniquely effective in clearing intrahepatic triglycerides, although long‐term biopsy data confirming fibrosis regression remain pending [85].

### 6.3 Liver‐Directed Agents and Fibroblast Growth Factor 21 (FGF‐21) Analogs

Resmetirom, an oral and highly selective thyroid hormone receptor‐beta agonist, is the first and currently only FDA‐approved medication specifically indicated for MASH with moderate‐to‐advanced liver fibrosis (F2–F3) [86]. In the pivotal phase 3 MAESTRO‐NASH trial, resmetirom demonstrated significant, biopsy‐confirmed steatohepatitis resolution and structural fibrosis improvement by accelerating hepatic lipid metabolism. FGF‐21 analogs, such as efruxifermin and pegozafermin, directly target hepatic metabolism to drastically reduce lipotoxicity [87]. Efruxifermin has shown highly promising, biopsy‐confirmed improvements in both steatosis and actual fibrosis regression specifically in F2–F3 patients, presenting a potent direct antifibrotic mechanism [88]. Legacy drugs like pioglitazone, a PPAR‐gamma agonist, can improve MASH histology; however, they are severely constrained by adverse side effects, including significant weight gain, fluid retention, and bone density reduction [64].

### 6.4 Central Nervous System Agents and Amylin Analogs

Combinations like phentermine/topiramate (acting via sympathomimetic mechanisms and GABA modulation) or naltrexone/bupropion (acting via opioid antagonism and dopamine/norepinephrine reuptake inhibition) effectively promote 5%–9% weight loss by significantly suppressing appetite [89]. However, these agents lack specific histologic outcome data related to MASLD and require highly cautious monitoring due to potential neuropsychiatric side effects and hepatotoxicity [90]. CagriSema, a co‐formulation of the amylin analog cagrilintide and semaglutide, presents significant synergistic potential for weight loss, although explicit trials are still needed to validate its safety in advanced MASH [91].

### 6.5 Considerations in Advanced Liver Disease

GLP‐1 RAs have demonstrated safety and considerable benefits in patients with compensated cirrhosis (Child‐Pugh A), significantly lowering the rates of hepatic decompensation and hepatocellular carcinoma [92]. However, their use in decompensated cirrhosis (Child‐Pugh B/C) remains contentious due to the heightened risks of sarcopenia, exacerbated malnutrition from delayed gastric emptying, and altered drug pharmacokinetics in cases of severe hepatic impairment.

## 7 Conclusion and Future Directions

MASLD is a highly complex, multisystemic epidemic fundamentally rooted in systemic IR, severe lipotoxicity, and significant disruptions in the microbiome and gut–liver axis. Although comprehensive lifestyle modifications and significant, sustained weight loss are critical foundations of conservative management, the therapeutic landscape has evolved markedly over the past 3 years. The recent FDA approval of resmetirom offers a targeted, highly specific option for patients with advanced fibrotic MASH. Simultaneously, the groundbreaking introduction of multireceptor incretin agonists (GLP‐1/GIP/glucagon) provides unprecedented, nonsurgical approaches to fully resolve steatohepatitis and halt fibrotic progression through substantial weight loss and restoration of insulin sensitivity.

Looking ahead, the validation of highly accurate noninvasive diagnostic methods for pediatric populations, the acquisition of long‐term safety data for novel therapies in advanced cirrhosis, and the resolution of the significant costs and global health equity challenges associated with these medications are urgent clinical priorities for the global hepatology community. Effectively addressing the global MASLD epidemic necessitates a highly personalized, multidisciplinary approach that focuses on aggressively mitigating cardiometabolic mortality while directly reversing hepatic fibrogenesis.

## Funding

This study was partially supported by the National Research Foundation of Korea (NRF) grant funded by the Korean government (MSIT) (RS‐2024‐00455210) and the Research Supporting Program of the Korean Association for the Study of the Liver (KASL2025‐01).

## Conflicts of Interest

The author declares no conflicts of interest.

## Data Availability

Data sharing is not applicable to this article as no datasets were generated or analysed during the current study.
